# Supplementary material for: Associations of the volume and proportion of vigorous-intensity physical activity with all-cause, cardiovascular, and cancer mortality: a systematic review and meta-analysis
Source: PeerJ. 2025 Jun 4;13:e19538. doi: 10.7717/peerj.19538 (PMC12145088; doi:10.7717/peerj.19538)
Supplement: Supplemental Information 2 [file peerj-13-19538-s002.docx]

**Systematic Review and Meta-Analysis Rationale for “The effects of vigorous-intensity physical activity volume and proportion on all-cause, cardiovascular, and cancer mortality: a systematic review and meta-analysis”**

1. **The rationale for conducting the systematic review and meta-analysis**

The rationale for conducting this systematic review and meta-analysis stems from several key gaps and uncertainties in the existing literature regarding vigorous-intensity physical activity (VPA) and its effects on mortality risks.

First, previous studies have varied in the amount of VPA examined, which makes it difficult to determine the optimal volume of VPA for reducing mortality risks. Understanding the effects of VPA volume on health benefits and finding the optimal volume for maximizing the health benefits is essential for forming clearer public health guidelines. While some studies suggest that increasing VPA volume results in greater health benefits, others indicate that the relationship may plateau or even diminish after a certain threshold. Thus, it is important to investigate whether there is a dose-response relationship between the volume of VPA and mortality risk, and if so, to identify the optimal volume of VPA needed to achieve substantial mortality risk reduction.

Another important issue is the proportion of VPA within total moderate-to-vigorous physical activity (MVPA). While guidelines recommend specific volumes of VPA or MVPA, the ideal proportion of VPA to MVPA for reducing mortality risks remains unclear. Some studies suggest that a higher proportion of VPA within MVPA may be beneficial up to a certain point, but the optimal range has not been well-defined.

Therefore, this review aims to synthesize evidence from large-scale observational studies to provide a more comprehensive understanding of how VPA volume and its proportion to MVPA impact mortality risks. The findings could inform future physical activity guidelines and recommendations for the general population, ultimately promoting better health outcomes.

1. **The contribution that it makes to knowledge in light of previously published related reports, including other meta-analyses and systematic reviews.**

This study makes a significant contribution to existing knowledge by providing a detailed analysis of the effects of VPA volume and its proportion within MVPA on all-cause, cardiovascular disease (CVD), and cancer mortality. Compared to prior meta-analyses and systematic reviews, several key advancements are made:

Dose-Response Relationship for VPA Volume

Previous studies have generally examined the relationship between physical activity and mortality but often did not clearly define the optimal volume of VPA for health benefits. This study identifies a curvilinear inverse dose-response relationship, showing that 180 minutes of VPA per week offers substantial mortality risk reduction, with further increases in VPA yielding only modest additional benefits. This provides more specific guidance on the amount of VPA required for maximum benefit.

Proportion of VPA to MVPA

While earlier reviews have explored the benefits of VPA and MVPA separately, this study uniquely assesses how the proportion of VPA within MVPA influences mortality risks. It finds a U-shaped inverse relationship, demonstrating that a 30-60% VPA proportion within MVPA is most effective in reducing all-cause and CVD mortality. This insight refines existing guidelines by highlighting the optimal balance between VPA and MPA.

In summary, this meta-analysis provides clearer, evidence-backed recommendations for public health interventions, emphasizing the importance of engaging in at least 180 minutes of VPA per week and ensuring that 30-60% of MVPA consists of VPA. These findings refine and expand upon earlier recommendations, offering practical guidance for maximizing health benefits through physical activity.

1. **Description: Who It Is Intended For**

This systematic review and meta-analysis is intended for public health researchers, policymakers, and health professionals who are responsible for developing and refining physical activity guidelines. It is also valuable for exercise scientists and epidemiologists interested in understanding the relationship between vigorous-intensity physical activity (VPA) and mortality outcomes.

The findings provide clear evidence-based recommendations for optimal VPA volumes and their proportion to moderate-to-vigorous physical activity (MVPA), aiding professionals in creating precise, actionable strategies to promote better health outcomes. Additionally, the study is relevant for educators and advocates aiming to enhance public awareness and implementation of evidence-based physical activity practices.
