# Supplementary material for: Associations of the volume and proportion of vigorous-intensity physical activity with all-cause, cardiovascular, and cancer mortality: a systematic review and meta-analysis
Source: PeerJ. 2025 Jun 4;13:e19538. doi: 10.7717/peerj.19538 (PMC12145088; doi:10.7717/peerj.19538)
Supplement: Supplemental Information 3 — These data were derived from the studies included in this analysis. [file peerj-13-19538-s003.docx]

**Appendix**

The Appendix contains

- eTable 1 (Details of the literature search)

- eTable 2 (Quality assessments)

- eTable 3 (Calculations of minutes/week of VPA)

- eTable 4,5 (Ascertainments and calculations of the proportion of VPA to MVPA)

- eTable 6 (Characteristics of included studies)

- eTable 7,8 (Results of dose-response meta-analyses)

- eFigure 1 (Quality assessment of included non-randomized studies evaluated by the ROBINS-I tool)

- eFigure 2 (Funnel plots for varying volumes of VPA dose-response analysis)

- eFigure 3 (Decorrelated residuals-versus-exposure plots for varying volumes of VPA dose-response analysis)

- eFigure 4-6 (Funnel plots for overall summary HRs for the highest volume of VPA vs. LMPA)

- eFigure 7-9 (Sensitivity analysis omitting one study at a time)

- eFigure 10-12 (Subgroup analysis of overall summary HRs for all-cause, CVD, and cancer mortality)

- eFigure 13 (Funnel plots for the proportion of VPA to MVPA dose-response analysis)

- eFigure 14 (Decorrelated residuals-versus-exposure plots for proportion of VPA to MVPA dose-response analysis)

- eFigure 15 (Funnel plots for overall summary HRs for the highest proportion of VPA to MVPA vs. the lowest)

**eTable 1. Literature search**

| **Web of Science** |
| --- |
| # Database: All Databases  # Searches:  #1: ((((((((((TS=(human)) OR TS=(humans)) OR TS=(female)) OR TS=(females)) OR TS=(male)) OR TS=(males)) OR TS=(*adult)) OR TS=(*adults)) OR TS=(*women)) OR TS=(*men)) OR TS=(aged) and Preprint Citation Index (Exclude – Database)  #2: (((((((((((((((TS=("vigorous physical activity")) OR TS=(vigorous)) OR TS=(VPA)) OR TS=("moderate to vigorous physical activity")) OR TS=(MVPA)) OR TS=("vigorous exercise"))OR TS=("high intensity physical activity")) OR TS=("vigorous leisure time physical activity")) OR TS=("vigorous physical activity"))) OR TS=(VLTPA)) OR TS=(high-tensity)) OR TS=(vigorous-intensity)) OR TS=("heavy intensity"))AND (((((TS=(moderate)) OR TS=("moderate intensity")) OR TS=("low intensity")) OR TS=("moderate continuous training")) OR TS=("light-intensity"))) and Preprint Citation Index (Exclude –– Database)  #3: (((((((((((TS=(mortality)) OR TS=("cardiovascular mortality")) OR TS=("All-cause mortality“)) OR TS=("cancer-mortality"))) OR TS=(death)) OR TS=(dead)) OR TS=(dying)) OR TS =(fatality)) OR TS=(mortalities)) OR TS=("CVD mortality")) and Preprint Citation Index (Exclude – Database)  #4: ((((((((((((TS=(cohort )) OR TS=(cohort analysis)) OR TS=(longitudinal study)) OR TS=(prospective study)) OR TS=(follow up))) OR TS=(prospective studies)) OR TS=(prospective cohort)) OR TS=(prospective cohorts)) OR TS=(case cohort)) OR TS=(nested case-control)) OR TS=(follow up studies)) OR TS=(follow-up study) and Preprint Citation Index (Exclude – Database)  5: #4 AND #3 AND #2 AND #1 and Preprint Citation Index (Exclude – Database)  Date Run: Fri Mar 22 2024 Results:1185 |
| **PubMed** |
| PubMed:  (humans[MeSH Terms] OR human[All Fields] OR humans[All Fields] OR female[MeSH Terms] OR female[All Fields] OR females[All Fields] OR male[All Fields] OR males[All Fields] OR male[MeSH Terms] OR aged[All Fields] OR adult[All Fields] OR adults[All Fields] OR adulthood[All Fields] OR adult[MeSH Terms])  AND (((((((((("vigorous physical activity") OR ("vigorous intensity")) OR ("VPA")) OR ("moderate to vigorous physical activity")) OR ("vigorous exercise")) OR ("VLTPA")) OR (" VPA"))) OR ("high intensity")) OR ("heavy intensity")) OR (vigorous [Title/Abstract])  AND (((("moderate intensity") OR (moderate[Title/Abstract])) OR ("low intensity"[Title/Abstract])) OR ("moderate continuous training")) OR ("light intensity")  AND ((((((((((((mortality)) OR (mortality [MeSH Terms])) OR (mortalities)) OR (death)) OR (dead)) OR (dying)) OR (fatality)) OR "CVD mortality")) OR ("cardiovascular mortality")) OR ("cancer mortality")) OR ("All-cause mortality") OR (death [MeSH Terms])  AND ("prospective study"[All Fields] OR "prospective studies"[All Fields] OR "prospective cohort"[All Fields] OR "prospective cohorts"[All Fields] OR "case cohort"[All Fields] OR "nested case-control"[All Fields] OR "longitudinal study"[All Fields] OR "longitudinal studies"[All Fields] OR "follow-up study"[All Fields] OR "follow up studies"[All Fields] OR "longitudinal studies"[MeSH Terms])  Results:342 |
| **Embase** |
| Embase  'mortality'/exp OR mortality OR 'cardiovascular mortality'/exp OR 'cardiovascular mortality' OR (('cardiovascular'/exp OR cardiovascular) AND ('mortality'/exp OR mortality)) OR 'all cause mortality'/exp OR 'all cause mortality' OR 'cancer mortality'/exp OR 'cancer mortality' AND 'follow up'/exp OR 'follow up' OR 'cohort analysis'/exp OR 'cohort analysis' OR 'prospective study'/exp OR 'prospective study AND ('vigorous physical activity'/exp OR 'vigorous physical activity' OR vpa:ab,ti OR mvpa:ab,ti OR 'moderate to vigorous physical activity'/exp OR 'moderate to vigorous physical activity' OR 'vigorous exercise'/exp OR 'vigorous exercise' OR 'high tensity*') AND ('moderate' OR 'light intensity')  Results:317 |

**eeTable 2. Newcastle-Ottawa Study (NOS) Quality Assessment (n=20)**

| Study | Selection | | | Comparability | Outcome | | | Total  stars |
| --- | --- | --- | --- | --- | --- | --- | --- | --- |
|  | Selection of the non-exposed cohort | Ascertainment of exposure | Demonstration  that outcome of interest was not present at the start of the study | Comparability  of cohorts on  the basis of the design or analysis | Assessment of outcome | Was follow-up long enough for outcomes to occur | Adequacy of follow-up of cohorts |  |
| Duarte 2024 (2) | \ | ⭐ | ⭐ | ⭐_ | ⭐ | \ | ⭐ | 5 |
| Mu 2022 (3) | ⭐ | ⭐ | ⭐ | ⭐⭐ | ⭐ | ⭐ | ⭐ | 8 |
| Lee 2022 (4) | ⭐ | ⭐ | ⭐ | ⭐_ | ⭐ | ⭐ | ⭐ | 7 |
| Ding 2022 (8) | ⭐ | ⭐ | ⭐ | ⭐_ | ⭐ | ⭐ | ⭐ | 7 |
| Wang 2021 (5) | ⭐ | ⭐ | ⭐ | ⭐_ | ⭐ | ⭐ | ⭐ | 7 |
| Zhao 2019 (6) | ⭐ | ⭐ | ⭐ | ⭐ | ⭐ | \ | ⭐ | 6 |
| Lopez 2019 (7) | ⭐ | ⭐ | ⭐ | ⭐ | ⭐ | \ | ⭐ | 6 |
| Kikuchi 2018 (9) | ⭐ | ⭐ | ⭐ | ⭐_ | ⭐ | ⭐ | ⭐ | 7 |
| Evenson 2016 (10) | ⭐ | ⭐ | ⭐ | ⭐_ | ⭐ | \ | ⭐ | 6 |
| Gebel 2015 (11) | ⭐ | ⭐ | \ | ⭐ | ⭐ | \ | ⭐ | 6 |
| Arem 2015 (12) | \ | ⭐ | ⭐ | ⭐⭐ | ⭐ | ⭐ | ⭐ | 7 |
| Shiroma 2014 (13) | ⭐ | ⭐ | ⭐ | ⭐ | ⭐ | ⭐ | ⭐ | 7 |
| Lahti 2014 (14) | ⭐ | ⭐ | ⭐ | ⭐ | ⭐ | ⭐ | \ | 6 |
| Wen 2011 (15) | ⭐ | ⭐ | \ | ⭐_ | ⭐ | \ | ⭐ | 5 |
| Leitzmann 2007 (16) | ⭐ | ⭐ | ⭐ | ⭐_ | ⭐ | \ | ⭐ | 6 |
| Yu 2003 (17) | ⭐ | ⭐ | ⭐ | ⭐_ | ⭐ | ⭐ | ⭐ | 7 |
| Rockhill 2001 (18) | ⭐ | ⭐ | ⭐ | ⭐ | ⭐ | \ | ⭐ | 6 |
| Lee 2000 (19) | ⭐ | ⭐ | ⭐ | ⭐ | ⭐ | ⭐ | ⭐ | 7 |
| Kushi 1997 (20) | ⭐ | ⭐ | ⭐ | ⭐ | ⭐ | \ | ⭐ | 6 |
| Lee 1995(21) | ⭐ | ⭐ | ⭐ | ⭐ | ⭐ | ⭐ | ⭐ | 7 |

Two authors (D.W. and L.Y.) applied a modified version of the Newcastle-Ottawa Quality Assessment Scale to evaluate the quality of the included studies. Informed by the methodological considerations specific to our research question, we predefined criteria for each item, and studies meeting these criteria were awarded one star. The criteria are as follows: (1) Selection of the non-exposed cohort: a star was awarded if participants in different intensity categories of physical activity were drawn from the same population; (2) Ascertainment of exposure: a star was awarded if physical activity was assessed using validated physical activity questionnaires or by trained interviewers; (3) Demonstration that the outcome of interest was not present at baseline: a star was awarded if participants with CVD, cancer, or other chronic diseases were excluded at baseline, adjusted for in the analysis, or if sensitivity analyses showed baseline conditions did not materially affect the estimates; (4) Comparability of cohorts on the basis of the design or analysis: a star was awarded if estimates were adjusted for age, gender (if applicable), smoking, alcohol consumption, and other components of physical activity (e.g., intensity). Two stars were given if the estimates were further adjusted for family history of cancer or CVD and socioeconomic status; (5) Assessment of outcome: a star was awarded if outcomes were confirmed through registers, death certificates, or next-of-kin reports (for all-cause mortality); (6) Was follow-up long enough for outcomes to occur: a star was awarded if the mean or median follow-up period was at least 10 years; and (7) Adequacy of follow-up of cohorts: a star was awarded if the number of participants lost to follow-up was ≤20% or if no significant differences were reported between those lost and followed. As we focused on internal validity rather than external validity, we excluded the "representativeness of the exposed cohorts" item from the original scale. Therefore, a maximum of 8 stars could be awarded. “-” denote one potential star not given, “\” indicates that no star would be awarded.

**eTable 3. Calculated minutes/week of VPA and original exposure categories (n=8)**

The quantification of varying volumes of VPA exposures was calculated using the midpoint between the lower and upper limit ranges. For open categories, we assumed that the width was the same as that of the adjacent category.

| First author, year of publication | Midpoints/means (preferred) or the range of the original  physical activities | Our calculation of minutes/week of VPA |
| --- | --- | --- |
| Lee 2022 | 1-74,75-149,150-224,225-299,300-374,375-449,450-599,>600 min/week of VPA | 37, 112, 187, 262, 337, 412, 525, 675 |
| Ding 2022 | 10-75,76-150, >150 min/week of VPA | 43, 113, 187 |
| Wang 2021 | 1-74,75-149,>=150 min/week of VPA | 37, 112, 187 |
| Zhao 2019 | 10–59, 60–149, 150–299, 300–599 and >=600 minutes/week | 35, 105, 225, 450, 750 |
| Arem 2015 | 0.1 to <7.5, 7.5 to <15, 15.0 to <30.0, >=30.0 MET h/week, leisure time VPA | 37, 112, 225, 375 |
| Wen 2011 | VPA volume: little (3·75–7·49 MET-h), Medium (7·50–16·49 MET-h), High (16·50–25·49 MET-h), Very high (>=25·50 MET-h) | 56, 120, 210, 300 |
| Leitzmann 2007 | Frequency of vigorous exercise: ＜1 ,1-3,4-7,＞7 times per week | 30, 120, 330, 510 |
| Kushi 1997 | Frequency of VPA: 1 time/week to a few times/month,2-4 times/week, >4 times/week. | 30, 180, 300 |

**eTable 4.** **Ascertainments and calculations of the proportion of VPA to MVPA for studies included in the dose-response meta-analysis for all-cause mortality**

The asterisk (*) indicates that the original article did not include information on person-years. The distributions of person-years were estimated based on the total number of cases, participants, and the follow-up period, as previously described [1]. For quantification of exposure, if the proportion of MVPA classified as VPA was reported as a range, the midpoint between the lower and upper limits was used.

| study | VPA ascertainment | proportion of VPA (to MVPA) ascertainment | cases | person-year | quantification of the exposure (The proportion of VPA to MVPA) | dose | response (HR/RR) 95% CI | | |
| --- | --- | --- | --- | --- | --- | --- | --- | --- | --- |
| Mu  2022 | VPA was defined as  physical activity of 6.0  METs or more such as running. | Same as Gebel 2015 | 58 | Per 10000 | 0% | 0 | 1 | 1 | 1 |
|  |  |  | 41 | Per 10000 | > 0% to ≤ 30% | 0.15 | 0.81 | 0.78 | 0.84 |
|  |  |  | 37 | Per 10000 | > 30% | 0.65 | 0.82 | 0.79 | 0.85 |
| Lee 2022 | Vigorous  physical activity was  defined as physical  activities ≥6METs. | Same as Gebel 2015 | 1040 | 79608 | 0% | 0 | 1 | 1 | 1 |
|  |  |  | 12872 | 690378 | >0 to ≤25% | 0.125 | 0.86 | 0.8 | 0.91 |
|  |  |  | 15793 | 886158 | >25 to ≤50% | 0.375 | 0.86 | 0.81 | 0.92 |
|  |  |  | 12262 | 813631 | >50 to ≤75% | 0.625 | 0.85 | 0.8 | 0.91 |
|  |  |  | 5414 | 499299 | >75 to ≤100% | 0.875 | 0.81 | 0.76 | 0.87 |
| Wang 2021 | Vigorous exercise is defined as activities lasting for at least 10 minutes that cause heavy sweating or large increases in breathing or heart rate. | Same as Gebel 2015 | 9241 | 856871 | 0% | 0 | 1 | 1 | 1 |
|  |  |  | 632 | 128101 | >0 to ≤25% | 0.125 | 0.89 | 0.81 | 0.98 |
|  |  |  | 1071 | 251297 | >25 to ≤50% | 0.375 | 0.9 | 0.83 | 0.98 |
|  |  |  | 2100 | 525805 | >50 to ≤ 75% | 0.625 | 0.83 | 0.78 | 0.88 |
|  |  |  | 1413 | 384194 | >75 to ＜100% | 0.875 | 0.85 | 0.79 | 0.91 |
|  |  |  | 2148 | 416281 | ＝100% | 1 | 0.9 | 0.85 | 0.96 |
| Lopez 2019 | Vigorous physical  activity was defined as  physical activities ≥ 6METs. | Same as Gebel 2015 | 4477 | 384868* | 0% | 0 | 1 | 1 | 1 |
|  |  |  | 292 | 26898* | >0% to <60% | 0.3 | 0.8 | 0.7 | 0.9 |
|  |  |  | 295 | 23977* | ≥60%§ | 0.8 | 0.89 | 0.79 | 1.01 |

**eTable 4** **Ascertainments and calculations of the proportion of VPA to MVPA for studies included in the dose-response meta-analysis for all-cause mortality, continued.**

| study | VPA ascertainment | the proportion of VPA ascertainment | cases | person-year | quantification of the exposure (The proportion of VPA to MVPA) | dose | response (HR/RR) 95% CI | | |
| --- | --- | --- | --- | --- | --- | --- | --- | --- | --- |
| Kikuchi men 2018 | Vigorous was defined  as physical activities ≥ 6METs. | Same as Gebel 2015 | 709 | 51019 | 0% | 0 | 1 | 1 | 1 |
|  |  |  | 426 | 41711 | > 0% to ≤ 30% | 0.15 | 0.99 | 0.87 | 1.13 |
|  |  |  | 160 | 21418 | ≥30% | 0.65 | 1.01 | 0.84 | 1.22 |
| Kikuchi women 2018 |  |  | 378 | 69327 | 0% | 0 | 1 | 1 | 1 |
|  |  |  | 225 | 50789 | > 0% to ≤ 30% | 0.15 | 1.03 | 0.86 | 1.24 |
|  |  |  | 88 | 26765 | ≥30% | 0.65 | 1.04 | 0.81 | 1.33 |
| Gebel 2015 | Vigorous activity is defined as activity that makes you breathe  harder or puff and pant, but not household chores. | To calculate the proportion of VPA to MVPA, multiply the duration of VPA (in minutes) by 2 and add the duration of MPA to obtain the total MVPA. Then divide the duration of VPA by the total MVPA | 4344 | 947294***** | 0% | 0 | 1 | 1 | 1 |
|  |  |  | 785 | 192319***** | > 0% to ≤ 30% | 0.15 | 0.89 | 0.81 | 0.98 |
|  |  |  | 1204 | 305168***** | ≥30% | 0.65 | 0.86 | 0.79 | 0.94 |
| Shiroma men 2014 | Vigorous was defined  as physical activities ≥ 6METs. | The percentage of MVPA performed at vigorous intensity was calculated as the VPA energy expenditure divided by MVPA energy expenditure (in METs) | 1407 | 47583 | ＜10% | 0.05 | 1 | 1 | 1 |
|  |  |  | 542 | 21223 | >10 to ≤ 25% | 0.175 | 0.92 | 0.81 | 1.06 |
|  |  |  | 543 | 22774 | >25 to ≤ 50% | 0.375 | 0.9 | 0.8 | 1.02 |
|  |  |  | 408 | 19691 | >50 to ≤ 75% | 0.625 | 0.96 | 0.84 | 1.09 |
|  |  |  | 561 | 25387 | >75% | 0.875 | 0.95 | 0.82 | 1.09 |
| Shiroma women 2014 |  |  | 1014 | 184265 | ＜10% | 0.5 | 1 | 1 | 1 |
|  |  |  | 362 | 83314 | >10 to ≤ 25% | 0.175 | 0.93 | 0.81 | 1.07 |
|  |  |  | 435 | 103803 | >25 to ≤ 50% | 0.375 | 1.03 | 0.9 | 1.17 |
|  |  |  | 374 | 91412 | >50 to ≤ 75% | 0.625 | 0.94 | 0.81 | 1.1 |
|  |  |  | 703 | 142390 | >75% | 0.875 | 1.26 | 1.14 | 1.4 |

**eTable 5. Ascertainments and calculations of the proportion of VPA to MVPA for studies included in the dose-response meta-analysis for CVD mortality**

The asterisk (*) indicates that the original article did not include information on person-years. The distributions of cases and person-years were estimated based on the total number of cases, participants, and the follow-up period, as previously described. For quantification of exposure, if the proportion of MVPA classified as VPA was reported as a range, the midpoint between the lower and upper limits was used.

| study | cases | person-years | quantification of the exposure (The proportion of VPA to MVPA) | dose | Response (HR/RR) 95% CI | | |
| --- | --- | --- | --- | --- | --- | --- | --- |
| Mu  2022 | 10 | Per 10000 | 0% | 0 | 1 | 1 | 1 |
|  | 7 | Per 10000 | > 0% to ≤ 30% | 0.15 | 0.77 | 0.7 | 0.84 |
|  | 7 | Per 10000 | > 30% | 0.65 | 0.86 | 0.77 | 0.96 |
| Lee  2022 | 269 | 80412 | 0% | 0 | 1 | 1 | 1 |
|  | 3084 | 702021 | >0 to ≤25% | 0.125 | 0.71 | 0.62 | 0.82 |
|  | 3754 | 900858 | >25 to ≤50%, | 0.375 | 0.75 | 0.66 | 0.86 |
|  | 2801 | 824999 | >50 to ≤75% | 0.625 | 0.74 | 0.64 | 0.85 |
|  | 1274 | 503989 | >75 to ≤100% | 0.875 | 0.73 | 0.63 | 0.84 |
| Wang 2021 | 1919 | 856871 | 0% | 0 | 1 | 1 | 1 |
|  | 116 | 128101 | >0 to ≤25% | 0.125 | 1.03 | 0.83 | 1.27 |
|  | 190 | 251297 | >25 to ≤50% | 0.375 | 1.02 | 0.85 | 1.22 |
|  | 346 | 525805 | >50 to ≤ 75% | 0.625 | 0.83 | 0.71 | 0.96 |
|  | 220 | 384194 | >75 to ＜100% | 0.875 | 0.83 | 0.7 | 0.99 |
|  | 414 | 416281 | ＝100% | 1 | 1.01 | 0.89 | 1.13 |
| Lopez  2019 | 1252 | 384868***** | 0% | 0 | 1 | 1 | 1 |
|  | 70 | 26898***** | >0% to <60% | 0.3 | 0.8 | 0.62 | 1.04 |
|  | 71 | 23977***** | ≥60% | 0.8 | 0.91 | 0.72 | 1.16 |
| Shiroma men 2014 | 429 | 47583 | ＜10% | 0.5 | 1 | 1 | 1 |
|  | 160 | 21223 | >10 to ≤ 25% | 0.175 | 1.06 | 0.85 | 1.34 |
|  | 168 | 22774 | >25 to ≤ 50% | 0.375 | 0.74 | 0.58 | 0.93 |
|  | 112 | 19691 | >50 to ≤ 75% | 0.625 | 0.79 | 0.6 | 1.02 |
|  | 183 | 25387 | >75% | 0.875 | 0.83 | 0.63 | 1.1 |
| Shiroma women 2014 | 225 | 184265 | ＜10% | 0.5 | 1 | 1 | 1 |
|  | 69 | 83314 | >10 to ≤ 25% | 0.175 | 0.86 | 0.62 | 1.19 |
|  | 64 | 103803 | >25 to ≤ 50% | 0.375 | 0.85 | 0.62 | 1.17 |
|  | 50 | 91412 | >50 to ≤ 75% | 0.625 | 0.87 | 0.61 | 1.24 |
|  | 142 | 142390 | >75% | 0.875 | 1.13 | 0.89 | 1.43 |

**Table 6. Characteristics of included studies (n=20)**

“‖” indicates that we reordered the study to improve visual aesthetics in the table.

| First author, year of publication | Number of study subjects, sex | Criteria for inclusion of subjects | Mean (SD) age or range (years | Cohort name, study period, country | Mean  follow- up years (unless otherwise stated) | Measurement of the exposure (period) | Covariates (maximally adjusted model) | Reference group | Main results (HR/RR) 95% CI, |
| --- | --- | --- | --- | --- | --- | --- | --- | --- | --- |
| Duarte 2024 [2] | 3,518 older adults (53.6%, women) | After excluding participants with cancer and CVD (n=6445) | 60–96 | Seniors-ENRICA-1cohorts, (2008-2022) Spanish | 11.65 ±2.79 (years) | PA was assessed with the EPIC questionnaire. (2008-2010) VPA: Cycling and sports | Age, sex, educational level, smoking status, alcohol consumption, BMI, cardiovascular disease, cancer, diabetes mellitus, hypertension, cohort, and additionally for the sum of time spent in all other physical activities | None Vigorous PA | categories: h/week of VPA All-cause mortality: 1.27 (0.54) mean (SD) h/week： 0.73 (0.59 0.92),  3.04 (0.92) mean (SD) h/week: 0.59 (0.46 0.77),  7.39 (3.42) mean (SD) h/week): 0.77 (0.60 0.99). |
|  | 3,273 older adults (53.1%, women) |  | 65–94 | Seniors-ENRICA-2 cohorts, (2015-2022) Spanish | 5.19 ±0.80 (years) | Questionnaire. PA was assessed with the EPIC questionnaire. (2015-2017) VPA: Cycling and sports |  |  |  |
| Mu 2022 [3] | 366,566 adults (53.9%, women) | Excluded pregnant women and those with baseline CVD or who reported no physical activity | 40–69 | UK biobank study (2006-2020) UK | 11.8 (median) | Questionnaire. Questionnaires adapted from the IPAQ short version (2006 - 2010). VPA was defined as a physical activity of 6.0 METs or more | Age, sex, education, income, race, Townsend index, smoking status, alcohol consumption, sedentary behavior, moderate-to-vigorous physical activity (MVPA), BMI, diet quality score, and family history of CVD | 0% of MVPA classified as VPA | categories: > 0% to ≤ 30%VPA/MVPA, > 30%VPA/MVPA: All-cause mortality(both) 0.81 (0.78–0.84),0.82 (0.79–0.85). CVD mortality(both) 0.77 (0.70–0.84),0.86 (0.77–0.96). All-cause mortality(men) 0.78 (0.75-0.82),0.81(0.78-0.85). All-cause mortality(women) 0.85 (0.81-0.90),0.84 (0.79-0.89). |
|  |  |  |  |  |  |  | Age, sex, education, income, race, Townsend index, smoking status, alcohol consumption, BMI, sedentary behavior, diet quality score and family history of CVD | 0 to < 150 min of MPA and 0 to < 75 min of VPA | Combinations of 150-300 mins/week of MPA with at least 150 mins/week of VPA reduce incident CVD risk (HR = 0.87, [95% CI 0.79–0.95]) and all-cause mortality (HR = 0.71, [95% CI 0.63–0.80]).  For incident HF and CVD mortality, 150-300 mins/week of MPA alongside 75-150 mins/week of VPA yield HRs of 0.77 (95% CI 0.65–0.92) and 0.72 (95% CI 0.57–0.93). |
| D.H. Lee 2022 [4] | 116 221 adults, (63%, women) | Excluded participants diagnosed with CVD or cancer and applying a 2-year lag time, the baseline is set to 1988 | 66 | Nurses’ Health Study and Health Professionals cohorts(1988-2018)US | 26(median) | A self-reported questionnaire in 1986 and updated every 2 years to report the average hours they spent during the past year on various activities. Up to 15 repeated measures of physical activity were available.Vigorous physical activity was defined as physical activities ≥6METs | Age, race, family history of CVD and cancer, postmenopausal hormone use, alcohol intake, total energy intake (quintiles), smoking status, sleep duration, Alternate Healthy Eating Index score, body mass index, vigorous physical activity (VPA), and moderate physical activity (MPA) were mutually adjusted | None Vigorous PA | categories:1-74,75-149,150-224,225-299,300-374,375-449,450-599,>600 min/wk, VPA  All-cause mortality:  0.87 (0.82–0.93),0.81 (0.76–0.87), 0.79 (0.74–0.85),0.77 (0.72–0.84), 0.78 (0.72–0.85),0.73 (0.65–0.81). 0.76 (0.68–0.85),0.74 (0.65–0.85). CVD mortality: 0.76 (0.67–0.86),0.69 (0.60–0.78), 0.73 (0.64–0.85),0.67 (0.57–0.79), 0.68 (0.56–0.82),0.65 (0.52–0.82), 0.67 (0.53–0.84),0.71 (0.54–0.93). |
|  |  |  |  |  |  |  | Age, race, family history of CVD and cancer, postmenopausal hormone use, alcohol intake, total energy intake (quintiles), smoking status, sleep duration, Alternate Healthy Eating Index score, body mass index, and total MVPA | 0% of MVPA classified as VPA | categories： >0 to ≤25%,>25 to ≤50%, >50 to ≤75%,>75 to ≤100%: All-cause mortality: 0.86 (0.80-0.91),0.86 (0.81-0.92), 0.85 (0.80-0.91),0.81 (0.76-0.87). CVD mortality: 0.71 (0.62-0.82),0.75 (0.66-0.86), 0.74 (0.64-0.85),0.73 (0.63-0.84). |
|  |  |  |  |  |  |  | Age, race, family history of CVD and cancer, postmenopausal hormone use, alcohol intake, total energy intake (quintiles), smoking status, sleep duration, Alternate Healthy Eating Index score, and body mass index | 0 min/wk  VPA and  MPA | Combinations of medium to high levels of VPA (75–300 min/week) and MPA (150–600 min/week) can yield nearly maximal mortality reduction (approximately 35%–42%). |
| Wang 2021 [5] | 403,681 individuals(51.7%,women) | Excluded those with disabilities or those unable to engage in moderate to vigorous physical activity, as well as those diagnosed with heart disease, stroke, or cancer at baseline | 42.8 (16.3) | National Health Interview Survey,(1997-2015)US | 10.1 (median) | 17 cross-sectional waves included physical activity questionnaires and information on covariates. Vigorous exercise is defined as activities lasting for at least 10 minutes that cause heavy sweating or large increases in breathing or heart rate.(1997-2013) | Age, sex, race/ethnicity, educational level, income, marital status, body mass index, smoking status, alcohol consumption, and MVPA | 0% of MVPA classified as VPA | categories:>0 to ≤25%,>25 to ≤50%,>50 to ≤75%,>75 to ＜100%＝100%. All-cause mortality: 0.89(0.81-0.98),0.90(0.83-0.98), 0.83(0.78-0.88),0.85(0.79-0.91), 0.90(0.85-0.96). CVD mortality:  1.03(0.83-1.27),1.02(0.85-1.22), 0.83(0.71-0.96),0.83(0.70-0.99), 1.01(0.89-1.13). cancer mortality 0.88(0.73-1.06),0.98(0.85-1.12), 0.80(0.72-0.90),0.89(0.77-1.02), 0.93(0.83-1.04). |
|  | | | | | | | Age, sex, race/ethnicity, education level, income, marital status, BMI, smoking status, alcohol consumption, and MPA | None Vigorous PA | categories： 1-74,75-149, ≥150 min/wk VPA all-cause mortality: 0.84(0.80,0.88),0.80(0.76,0.84), 0.76(0.72,0.79). CVD mortality: 0.92(0.82,1.03),0.79(0.70,0.91), 0.72(0.65,0.80) cancer mortality: 0.81(0.73,0.90),0.89(0.80,0.99), 0.83(0.76,0.90) |
|  |  |  |  |  |  |  | Age, sex, race/ethnicity, educational level, income, body mass index, smoking, and alcohol intake were assessed. Moderate intensity physical activity, VPA, and the interaction term were simultaneously included in a multivariable model | 0 min/wk  VPA and MPA | Combinations of 150 to 299 minutes per week of MPA along with 150 minutes per week or more of VPA suggested the lowest all-cause mortality risk among participants.  For CVD mortality, it was 300 minutes or more per week of MPA and 150 minutes per week or more of VPA.  For cancer mortality, it was 300 minutes per week or more of MPA and 1 to 74 minutes per week of VPA. |
| Zhao2019 [6] | 88,140participants (56%,women) | Excluded participants with less than 3 years of follow-up duration in 2009, aged under 40 years, the presence of chronic disease, missing information on PA or covariates, or the presence of pregnancy | 40–85 | National Health Interview Surveys (1997–2011)US | 9(median) | Twelve cross-sectional household interviews were conducted to gather health information, lifestyle behaviors, and data on the frequency and duration of leisure-time physical activity among sampled participants. (1997–2008) | Age, sex, race, education, marital status, body mass index, smoking, alcohol intake and mutually adjusted for both moderate- and vigorous-intensity activities | None Vigorous PA | categories: 10–59, 60–149, 150–299, 300–599 and ≥600 minutes/week. All-cause mortality: 0.74 (0.63-0.87),0.76 (0.68-0.86), 0.66 (0.59-0.75),0.69 (0.59-0.81), 0.58 (0.46-0.73). CVD mortality: 0.74 (0.50-1.10),0.91 (0.72-1.15),  0.67 (0.49-0.91),0.88 (0.65-1.21), 0.66 (0.40-1.07). cancer mortality: 0.79 (0.59-1.05),0.86 (0.71-1.04),  0.77 (0.63-0.95),0.67 (0.51-0.89), 0.61 (0.42-0.89). |
| Lopez 2019 [7] | 64,913 adults (56%, women) | Excluded individuals who reported no MVPA, and lacked information on covariates | 49.8 (13.6) | Health Survey for  England and the Scottish Health Survey (1994-2011) England and Scotland | 9.0± 3.6 （years） | Questionnaire. Once at baseline, physical activity was assessed via an interviewer-administered questionnaire, which captured data on sports and exercise including frequency, duration, and perceived intensity over the last 4 weeks, using a prompt card. Covariates were objectively measured. (1994-2008) | For all modalities and cancer mortality, alcohol consumption, total weighted volume of moderate to vigorous physical activity (MVPA), longstanding illness, body mass index (BMI), and psychological distress. For CVD, plus CVD diagnosis at baseline | 0% of MVPA classified as VPA | categories: >0% to <60%，≥60% VPA/MVPA All-cause mortality: 0.80 (0.70-0.90),0.89 (0.79-1.01). CVD mortality： 0.80 (0.62-1.04),0.91 (0.72-1.16). cancer mortality: 0.82 (0.67-0.99),0.95 (0.79-1.15). |
|  |  |  |  |  |  |  | Age, sex, education, and smoking, as well as alcohol consumption, total weighted volume of moderate-to-vigorous physical activity (MVPA), longstanding illness, cancer diagnosis at baseline, BMI, and psychological distress | moderate intensity PA | categories: Vigorous Intensity (excluding first 24 months)  All-cause mortality: 0.84 (0.76-0.93) CVD mortality： 0.86 (0.70-1.06) cancer mortality: 0.86 (0.73-1.02) |
| Ding2022‖ [8] | 346,627adults(54%,women) | Excluded participants diagnosed with CVD, cancer, degenerative neurological disorder, late-stage renal disease, chronic widespread pain, COPD and emphysema, or being underweight at baseline | 40–69 | UK biobank study(2007-2020)UK | 11.2(median) | Questionnaire.IPAQ regarding total minutes of walking, moderate physical activity (MPA), and VPA that lasted for at least 10 minutes at a time, in a typical week.(2007-2010) | Age, sex, educational attainment, marital status, country of birth, type of occupation, neighborhood deprivation, smoking status, alcohol intake, hypertension, diabetes, and diet quality index and MVPA. Models for VPA were further adjusted for MPA and walking | None Vigorous PA | categories:10-75,76-150, >150 (min/week, VPA): All-cause mortality: 0.87 (0.84-0.91),0.85 (0.80-0.89), 0.89 (0.84-0.94). CVD mortality: 0.75 (0.68-0.83),0.76 (0.67-0.86), 0.90 (0.81 - 1.02). cancer mortality: 0.94 (0.87-1.01),0.93 (0.84-1.02), 0.91 (0.83-1.00). |
| Kikuchi 2018 [9] | 83,454 adults (53.7%, women) | Excluded subjects with a history of cancer or cardiovascular disease, as well as those with physical limitations | M: 61.5(7.4) F: 62.0(7.6) | The Japan Public Health Center-based prospective study (2000-2012) Japan | 894,718 person-years | Questionnaire. The questionnaire administered once at baseline assessed the frequency, intensity, and duration of physical activity during leisure time. | Age, sex, public health centers, smoking, drinking, body mass index, diabetes history, hypertension status, and MVPA | 0% of MVPA classified as VPA | categories: >0% to <30%, ≥30% All-cause mortality: man:0.99 [0.87–1.13], 1.01 [0.84–1.22]. woman:1.03 [0.86–1.24], 1.04 [0.81–1.33]. |
| Evenson 2016 [10] | 3,809 adults (54.6%， women) | Excluded participants younger than 40 and classified as having prevalent conditions, those who died within the first two years of follow-up. | 55.3 | National Health and Nutrition Examination Survey cohort (2003-2011) US | 6.8 (median) | Both accelerometer-assessed physical activity (PA) and self-reported PA for one week. Vigorous physical activity was defined as physical activities ≥6METs. (2003-2006) | Age, sex, race, educational level, marital status, cigarette smoking, the interaction between current employment and follow-up time, need for special equipment to walk, arthritis, cancer, BMI, the interaction between body mass index categories and follow-up time, hypertension, diabetes, and other physical activity components | None Vigorous PA | categories: ≥ 0.1 minutes/day, Vigorous physical activity. accelerometer-assessed physical activity: all-cause mortality 1.02 (0.71, 1.47) CVD mortality 0.98 (0.50, 1.92) self-reported PA: all-cause mortality  0.69 (0.46, 1.02) CVD mortality  0.58 (0.27, 1.13) |
| Gebel 2015 [11] | 204,542 adults (55.2%, women) | Excluded adults older than 75 years, those who did not report any activity, and those with missing values for physical activity | 45–75 | SaxInstitute’s45 and Up study(2006-2014)Australia | 6.52±1.23(years) | Questionnaire.Once at baseline.Participants reported sessions (bouts ofat least 10 minutes) and the duration of walking and moderate-intensity and vigorous-intensity activities in the past week.(2006-2009) | Age, sex, education,marital status, urban/rural residence, smoking status, weight status,physical function, alcohol consumption, fruit andvegetable intake, and the total weighted volume of MVPA | 0% of MVPA classified as VPA | categories:> 0% to ＜30% VPA/MVPA, > 30%VPA/MVPA: all-cause mortality, man: 0.92 (0.84-1.02), 0.86 (0.79-0.94). women: 0.89 (0.76-1.01), 0.89 (0.80 0.99). both (n=164678)  0.89 (0.81-0.98),0.86 (0.79 to 0.94). |
| Arem  2015 [12] | 348,725 adults (68.8%, women) | Excluded individuals with missing BMI data or reporting a BMI of less than 15 or more than 60 | 62 (median) 21-98 (range) | National Cancer Institute Cohort Consortium includes 6 studies (1992-2014) US and Europe | 14.2 (median) | Questionnaire. The energy expended per activity is calculated by multiplying the estimated metabolic equivalent (MET) value by the number of hours per week and summed across activities to estimate overall leisure time physical activity (LTPA). (1992-2003) | Age, sex, smoking, alcohol use, educational level, marital status, history of cancer, history of heart disease, body mass index, and both moderate- and vigorous-intensity activities were mutually adjusted | None Vigorous LTPA | categories: 0.1 to <7.5, 7.5 to <15, 15.0 to <30.0, ≥30.0 MET h/week, LTPA Vigorous Level all sample:  0.80(0.78-0.83), 0.77(0.71-0.84), 0.78(0.73-0.83), 0.79(0.73-0.85). men: 0.78(0.75-0.82), 0.69(0.61-0.78), 0.72(0.66-0.79), 0.77(0.70-0.85). women:  0.83(0.79-0.88), 0.85(0.77-0.94), 0.86(0.78-0.94), 0.81(0.72-0.91). |
| Shiroma 2014 [13] | 46,650 adults (82.9%, women0 | Excluded women and men who were missing information on physical activity at baseline or who reported diagnoses of cardiovascular disease (CVD), cancer, or diabetes occurring before baseline | M:  66.1(7.7) F: 54.6(7.0) | Harvard Alumni Health Study, men (1988-2008). Women’s Health Study, women (1992-2012) US | M:17.3 (mean) F: 16.4 (mean) | Questionnaire. Participants self-reported the number of flights of stairs climbed daily, the number of blocks walked daily, and the frequency and duration spent doing sports or recreational activities. Physical activity questionnaires were administered three times in men (1988,1993,1998) and in women at baseline and every 2-3 years. (1992-2008) | M: Age, MVPA, smoking status, dietary factors, alcohol consumption, BMI, high cholesterol and hypertension F: Age, MVPA, BMI, high cholesterol, hypertension, clinical trial randomization, smoking status, dietary factors, alcohol consumption, postmenopausal status, hormone therapy, and parental history of myocardial infarction | ＜10% of MVPA classified as VPA | categories: >10% to <25%, >25% to ≤50%, >50% to ≤75%, >75% percent of MVPA performed at vigorous Intensity. all-cause mortality: Men: 0.92(0.81.1.06),0.90(0.80.1.02), 0.96(0.84.1.09),0.95(0.82.1.09). Women: 0.93(0.81,1.07),1.03(0.90,1.17), 0.94(0.81,1.10),1.26(1.14,1.40). CVD mortality: Men: 1.06 (0.85,1.34),0.74(0.58,0.93), 0.79 (0.60,1.02),0.83 (0.63,1.1). Women: 0.86 (0.62.1.19),0.85 (0.62, 1.17), 0.87 (0.61.1.24),1.13 (0.89, 1.43). |
| Lahti 2014 [14] | 6,429adultsboth | Excluded participants who were missing information on some study variables or reported no leisure-time physical activity | 40-60 | Helsinki Health Study(2000-2012)Finland | 12 (mean） | Questionnaire.Once at baseline, participants reported the time usually spent per week on physical activity during leisure time, including transportation, within the previous 12 months. Vigorous physical activity (e.g., jogging/running)  (2000-2002) | Age, gender, occupational social class, physical and mental strenuousness of work, smoking and drinking habits, body mass index, physical functioning, mental health, limiting longstanding illness, and volume of physical activity | Low moderate LTPA | categories: High moderate: 0.73 (0.49–1.11)  Vigorous LTPA all-cause mortality:  0.55 (0.32–0.94). |
| Wen  2011 [15] | 416,175 adults (52.1%, women) | \ | ≥20 | Historically prospective cohort study (1996-2008) Taiwan | 8.05±4.21 | Questionnaire.  Their leisure-time physical activity (LTPA) level was determined by three multiple-choice questions: types and intensities of weekly LTPAs in the past month, weekly duration spent on different LTPA, and physical activity at work. | Age, sex, education, physical labor at work, smoking, drinking, fasting blood glucose, systolic blood pressure, total cholesterol, BMI, diabetes, hypertension, and history of cancer | physical activity volume<3·75 MET-h per week | categories: vigorous intensity physical activity volume little (3·75–7·49 MET-h), Medium (7·50–16·49 MET-h), High (16·50–25·49 MET-h), Very high (≥25·50 MET-h). All-cause mortality: 0·73(0·54–0·98),0·67(0·57–0·78), 0·60(0·48–0·76),0·60(0·53–0·68). CVD mortality: 0·70 (0·31–1·57),0·56(0·37–0·86). 0·55 (0·32–0·94),0·54(0·41–0·72). cancer mortality: 0·77 (0·48–1·23),0·80(0·63–1·00), 0·80 (0·59–1·11),0·74(0·62–0·88). |
| Leitzmann 2007 [16] | 252,925 adults (44%, women) | Excluded individuals who reported a previous diagnosis of cancer, cardiovascular disease, or emphysema | 50-71 | NIH-AARP Diet and Health Study. (1995-2001) US | 1,265,347 person-years | Questionnaire. The baseline questionnaire inquired about structured vigorous exercise during the previous year. Within 6 months of the baseline questionnaire, the second questionnaire requested information on the average time spent each week on activities of at least moderate intensity. (1995-1996) | Age, sex, body mass index, smoking status, race/ethnicity, education level, marital status, family history of cancer, menopausal hormone therapy, aspirin use, multivitamin use, and intakes of vegetables, fruit, red meat, and alcohol. Activity of at least moderate intensity and vigorous exercise were mutually adjusted | None Vigorous PA | categories: ＜1,1-3,4-7,＞7 times per week, vigorous exercise. All-cause mortality: 0.77(0.71-0.83),0.77(0.72-0.82), 0.68(0.63-0.73),0.71(0.66-0.77) CVD mortality: 0.72(0.63-0.82),0.74 (0.66-0.84), 0.66(0.59-0.76),0.71 (0.62-0.82) cancer mortality: 0.91(0.81-1.02),0.94 (0.85-1.04), 0.82(0.74-0.92),0.95 (0.85-1.07). |
| Yu 2003 [17] | 1,975men | Excluded men with mainly symptomatic evidence of coronary heart disease (CHD), a doctor-diagnosed myocardial infarction, or grade 1 or grade 2 angina, those with probable ECG ischemias， and who died within two years | 49-64 | Caerphilly collaborative heart disease study. (1984-1997)UK | 10.5(mean) | A detailed questionnaire derived from the Minnesota Leisure Time Physical Activity (LTPA) questionnaire was used to estimate energy expenditure expressed as an activity index (AI) in kcal/day from a record of leisure activity during the preceding 12 months. The Health Insurance Plan questionnaire was used to assess physical activity at work. | Age, combined light and moderate intensity activity, diastolic blood pressure, and body mass index were considered continuous variables. Smoking status, social class, family history of coronary heart disease (CHD), history of diabetes mellitus in the past five years, and job physical activity class | Heavy activity tertile1 (0-0.06kcal/day) | The heavy activity index (AI) is calculated by summing activities with intensity codes above 6.0, including climbing stairs, swimming, and jogging. This index represents energy expenditure during physical activity. categories: Heavy activity tertile2 (0.7 23.8kcal/day), tertile3 (23.9-2142.9kcal/day) All-cause mortality: 0.87 (0.65-1.17),0.61 (0.43-0.86). CVD mortality: 0.86 (0.57-1.32),0.38 (0.21-0.67). |
| Rockhill 2001 [18] | 80,348 women | Excluded women at baseline with a history of cardiovascular disease or cancer, as well as those who died before 1988 | 34-75 | Nurses’ Health Study (1980-1996) US | 588,426 person-years | Questionnaire. In 1986, 1988, and 1992 Women were asked to report the average time spent per week during the previous year on activities such as walking and more vigorous (non-walking) physical activity, including running, heavy lifting, or participating in strenuous sports. | Age at baseline, smoking status, recent alcohol consumption, height, body mass index, and postmenopausal hormone use | Less than 1 hour per Week of Walking, and <1 hour of Vigorous Physical Activity/wk | Among women walking less than 1 hour per week, engaging in more than 1 hour per week of vigorous activity was linked to a moderate (approximately 20%–25%) reduction in mortality risk. Furthermore, those engaging in over 3 hours of vigorous physical activity per week and 3 or more hours of walking experienced the greatest mortality risk reduction (37%). |
| I-Min Lee 2000 [19] | 13,485 men | Excluded men reporting physician-diagnosed cardiovascular disease, cancer, or chronic obstructive pulmonary disease | 57.7 | Harvard Alumni Health Study (1977-1992) US | 1977-1992 (range) | Questionnaire. On the 1977 questionnaire, alumni reported the daily number of blocks walked and flights of stairs climbed. They also reported their sports or recreational activities during the past year, including the time spent per week and the weeks per year of participation for each activity. | Age, Quetelef's index, cigarette smoking, alcohol consumption, and early (<65 years) parental death. Additionally, each component of physical activity was adjusted for the other four components | Vigorous activities (＜630kJ /week) | categories: Vigorous activities: (630-＜1680kJ/week), (1680-＜3150kJ/week), (3150-＜6300kJ/week), (≥6300kJ/week). All-cause mortality: 0.89(0.77,1.02),0.82(0.70,0.96), 0.82(0.71,0.96),0.77(0.67,0.89). |
| Kushi 1997 [20] | 32,763 postmenopausal Iowa women | Excluded women with baseline diseases (self-report of any cancer excluding skin cancer, heart disease, angina, or heart attack); those who died within the first 3 years of follow-up; those who didn't respond to questions about covariates | 55-69 | The Iowa Women's Health Study.(1986-1992)US | 1986-1992(range) | Questionnaire.Participants were asked a general question about regular physical activity that has been used for over 3 decades by the Gallup poll. Secondly, participants completed 2 questions asking how often they participate in moderate physical activity and vigorous physical activity (e.g., jogging, racket sports, swimming, aerobics, or strenuous sports). | Age at baseline, age at menarche, age at menopause, age at first live birth, parity, alcohol Intake, total energy intake, smoking status, estrogen use, body mass Index at baseline, body mass Index at age 18 years, waist-to-hip ratio, first-degree female relative with cancer, high blood pressure, diabetes, education level, and marital status | None Vigorous PA | categories:Frequency of vigorous physical activity: 1 times/week to a few times/month,  2-4 times/week, >4 times/week. All-cause mortality: 0.89 (0.67-1.12),0.74 (0.52-1.05), 0.57 (0.31-1.07). CVD mortality: 0.85 (0.50-1.44),0.59 (0.28-1.25), 0.20 (0.03-1.41). cancer mortality: 1.09 (0.77-1.53),0.83 (0.52-1.33), 0.69 (0.31-1.54). |
| I-Min Lee 1995 [21] | 17,321 men | Excluded men with self-reported or physician-diagnosed cardiovascular disease, cancer, or chronic obstructive pulmonary disease | 46 | Harvard University alumni cohort. (1962-1988) US | 1962 or 1966 to1988 (range) | Questionnaire.  Physical activity was assessed by asking alumni about flights of stairs climbed, city blocks walked, types of sports or recreational activities engaged in, and the time (hours per week) spent on each of these sports and recreational activities in 1962 or 1966. | Age, Quetelet's index, cigarette habit, physician-diagnosed hypertension or diabetes mellitus, and early (<65 years) parental death. Relative risks for vigorous and non-vigorous energy expenditure is mutually adjusted | Vigorous activities (＜630 kJ/week) | categories: 630-＜1,680,1,680-＜3,150, 3,150-<6,300,≥6300 (kJ/week) All-cause mortality: (exclude the first five years after the physical activity assessment) 0.88(0.81-0.96),0.91(0.81-1.02), 0.87(0.76-1.00),0.86(0.76-0.96). |

**eTable 7. All-cause, CVD, and cancer mortality hazard ratios (95% CI) with 0 min/week of VPA as the reference level**

Dose-response relation between the volume of VPA (with 0 min/week of VPA as the reference) and HRs for all-cause, CVD, and cancer mortality, estimated with restricted cubic spline regression and generalized least square trend estimation for summarized dose-response data.

| dose (varying volumes of VPA) | 0 | 30 | 56 | 105 | 120 | 180 | 225 | 300 | 375 | 450 | 750 |
| --- | --- | --- | --- | --- | --- | --- | --- | --- | --- | --- | --- |
| all-cause mortality (HR)  95%CI | 1 | 0.95 | 0.9 | 0.83 | 0.82 | 0.78 | 0.77 | 0.77 | 0.77 | 0.77 | 0.79 |
|  |  | 0.94 to 0.95 | 0.89 to 0.91 | 0.82 to 0.84 | 0.8 to 0.83 | 0.76 to 0.79 | 0.75 to 0.78 | 0.75 to 0.78 | 0.75 to 0.79 | 0.75 to 0.8 | 0.75 to 0.84 |
| CVD mortality (HR) 95%CI | 1 | 0.94 | 0.9 | 0.82 | 0.81 | 0.77 | 0.76 | 0.76 | 0.76 | 0.78 | 0.81 |
|  |  | 0.93 to 0.95 | 0.88 to 0.92 | 0.79 to 0.85 | 0.77 to 0.84 | 0.73 to 0.8 | 0.72 to 0.79 | 0.73 to 0.79 | 0.73 to 0.8 | 0.73 to 0.84 | 0.71 to 0.93 |
| cancer mortality (HR) 95%CI | 1 | 0.96 | 0.93 | 0.89 | 0.88 | 0.86 | 0.86 | 0.85 | 0.85 | 0.85 | 0.84 |
|  |  | 0.95 to 0.97 | 0.91 to 0.95 | 0.85 to 0.92 | 0.84 to 0.91 | 0.82 to 0.9 | 0.82 to 0.89 | 0. 82 to 0.89 | 0.81 to 0.9 | 0.78 to 0.92 | 0.74 to 0.96 |

**eTable 8. All-cause and CVD mortality hazard ratios (95% CI) with 0% of VPA to MVPA as the reference level**

Dose-response relation between the proportion of VPA to MVPA (with 0% of VPA to MVPA as the reference level) and HRs for all-cause and CVD mortality, estimated with restricted cubic spline regression and generalized least square trend estimation for summarized dose-response data.

| dose (proportion of VPA to MVPA) | 0% | 5% | 12.5% | 15.0% | 17.5% | 30.0% | 37.5% | 65.0% | 80.0% | 87.5% | 100.0% |
| --- | --- | --- | --- | --- | --- | --- | --- | --- | --- | --- | --- |
| all-cause mortality (HR) 95%CI | 1 | 0.98 | 0.96 | 0.95 | 0.94 | 0.92 | 0.90 | 0.91 | 0.92 | 0.94 | 0.95 |
|  |  | 0.98 to 0.99 | 0.95 to 0.97 | 0.94 to 0.97 | 0.93 to 0.96 | 0.89 to 0.94 | 0.88 to 0.93 | 0.87 to 0.94 | 0.88 to 0.97 | 0.89 to 0.99 | 0.90 to 1.01 |
| CVD mortality (HR) 95%CI | 1 | 0.98 | 0.95 | 0.94 | 0.93 | 0.90 | 0.88 | 0.89 | 0.92 | 0.94 | 0.97 |
|  |  | 0.97 to 0.99 | 0.92 to 0.97 | 0.94 to 0.97 | 0.90 to 0.96 | 0.85 to 0.95 | 0.83 to 0.94 | 0.82 to 0.96 | 0.84 to 1 | 0.85 to 1.03 | 0.87 to 1.08 |

**eFigure 1. Quality assessment of included non-randomized studies evaluated by the ROBINS-I (Risk Of Bias In Non-randomized Studies - of Interventions) tool.**


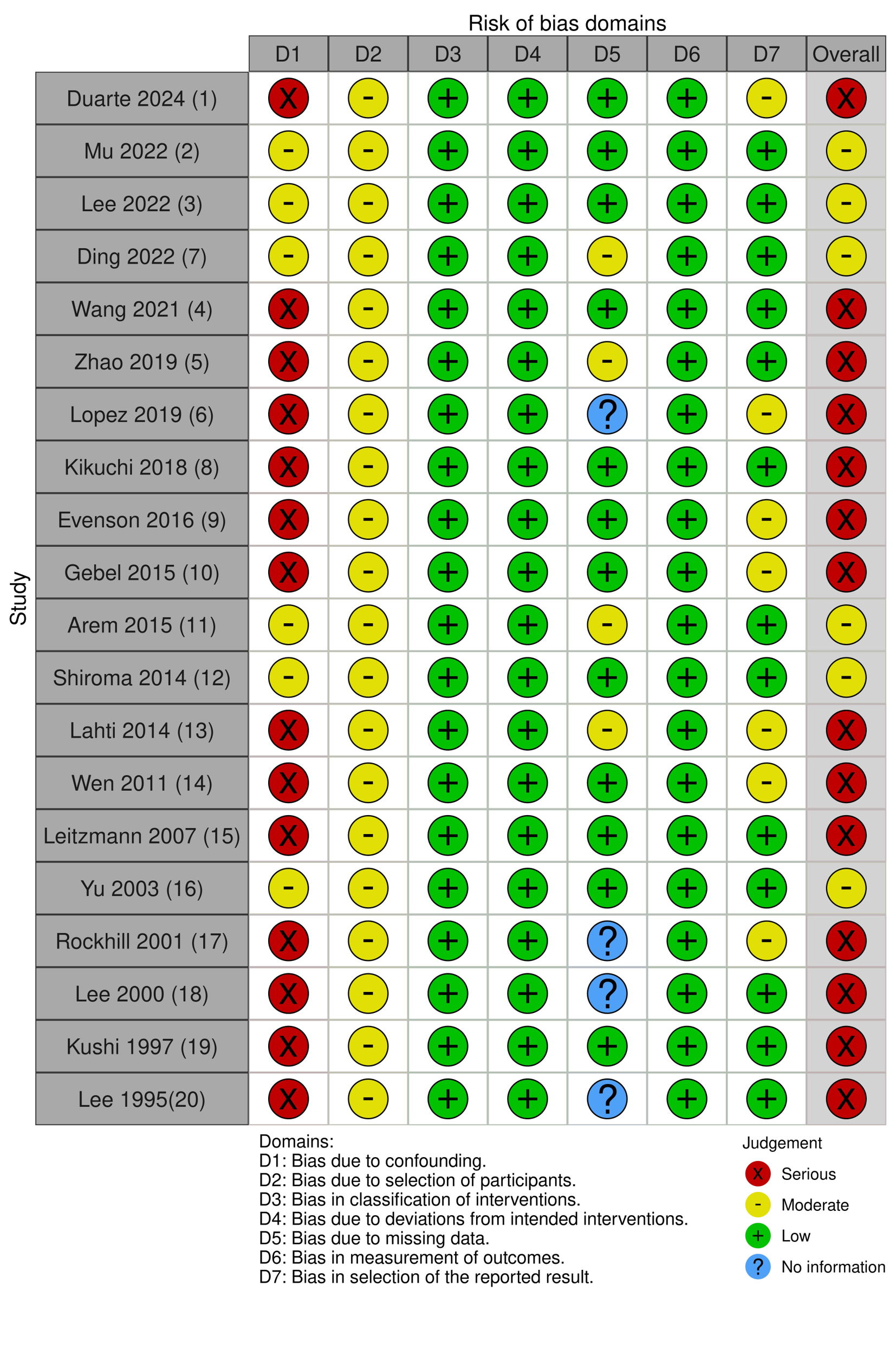


**
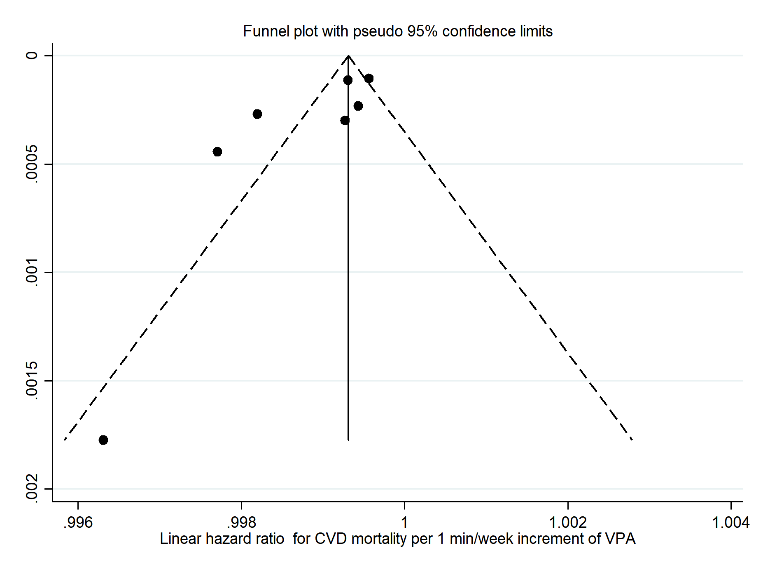

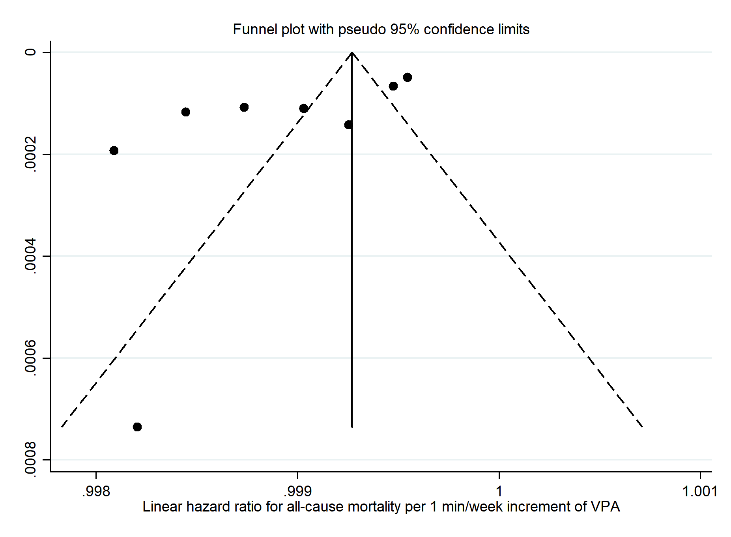
eFigure 2.** **Funnel plot for the linear association of the HR for all-cause, CVD, and cancer mortality risk against the standard error of the log (HR) per 1-minute/week increment of VPA**

**
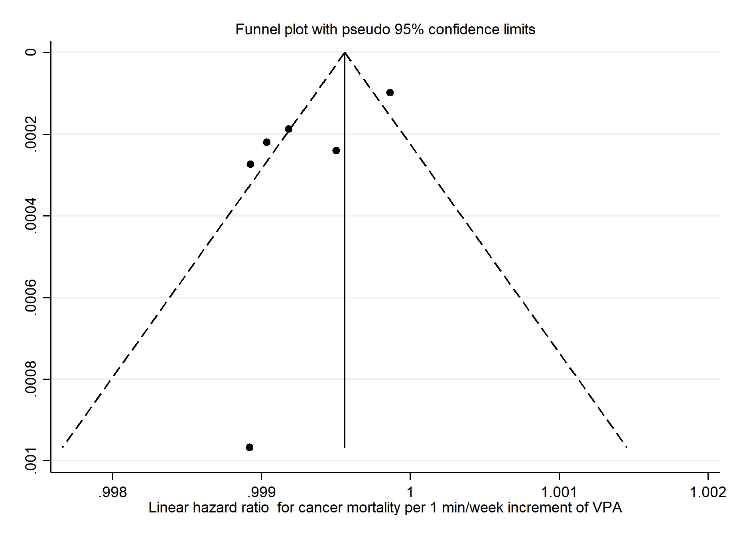
**

**
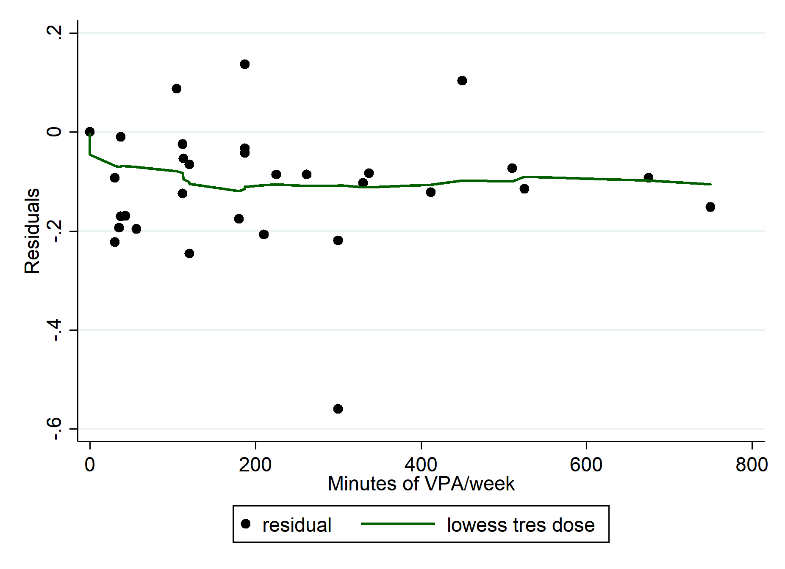

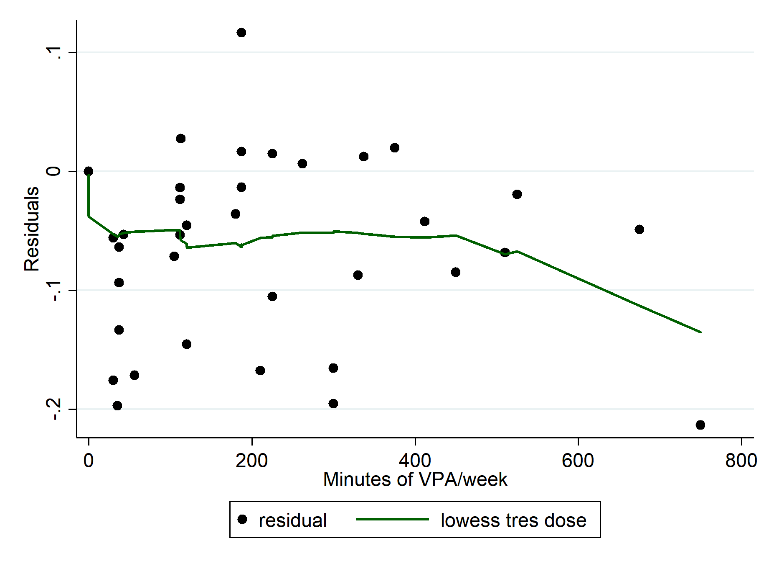
eFigure 3. Decorrelated residuals-versus-exposure plots for varying volumes of VPA dose-response analysis**

all-cause mortality, decorrelated residuals-versus-exposure plot CVD mortality, decorrelated residuals-versus-exposure plot


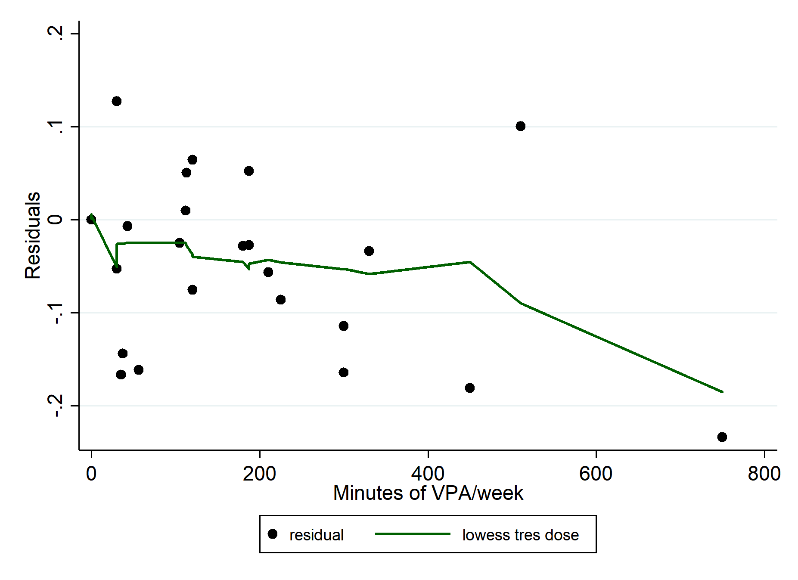


cancer mortality, decorrelated residuals-versus-exposure plot

**eFigure 4. Funnel plots for overall summary HRs for the highest volume of VPA vs. LMPA for all-cause mortality**

**
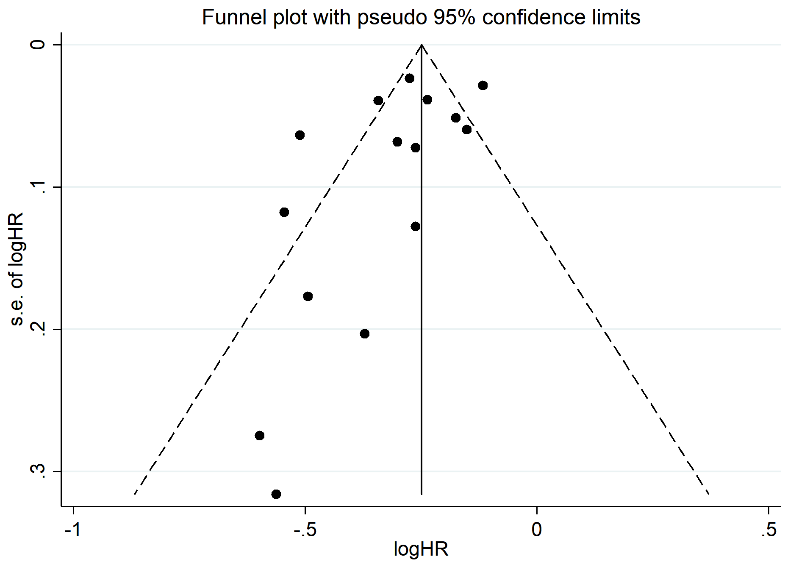
**

**eFigure 5. Funnel plot for overall summary HRs for the highest volume of VPA vs. LMPA for CVD mortality**

**
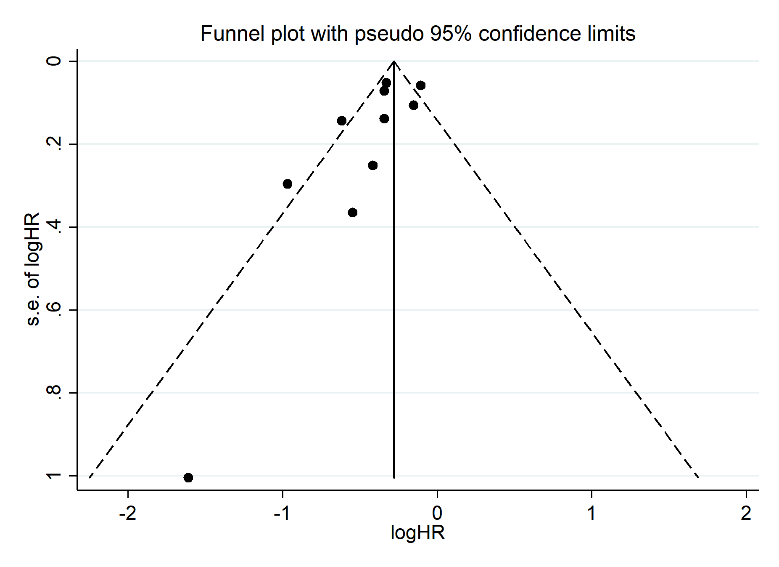
**

**eFigure 6. Funnel plot for overall summary HRs for the highest volume of VPA vs. LMPA for cancer mortality**

**
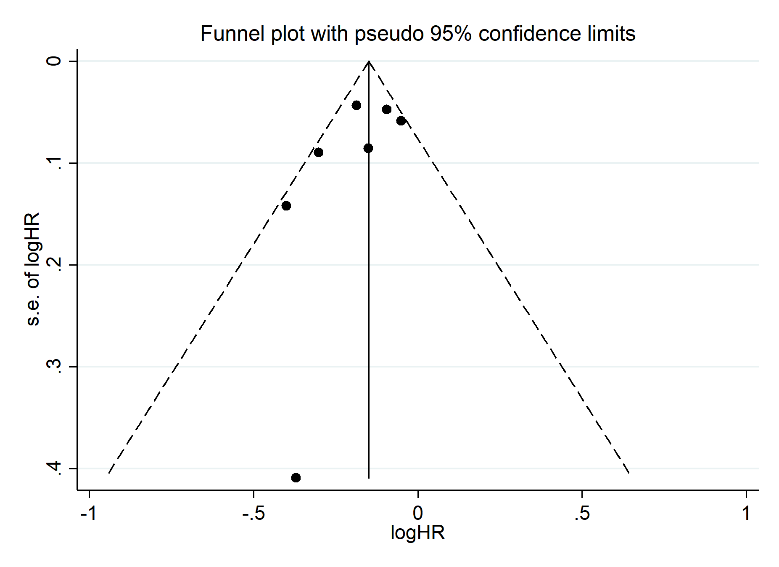
**

**eFigure 7. Sensitivity analysis for the overall summary hazard ratios for all-cause mortality, omitting one study at a time.**

**
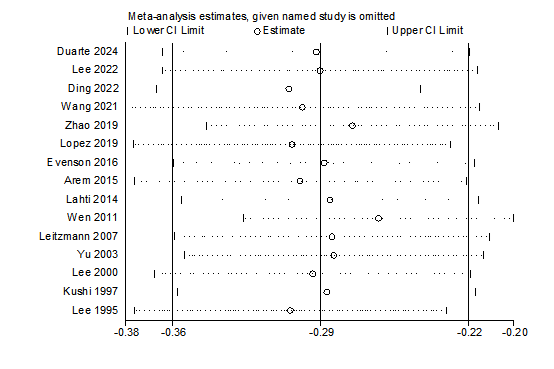
**

**eFigure 8. Sensitivity analysis for the overall summary hazard ratios (HRs) for CVD mortality, omitting one study at a time.**

**
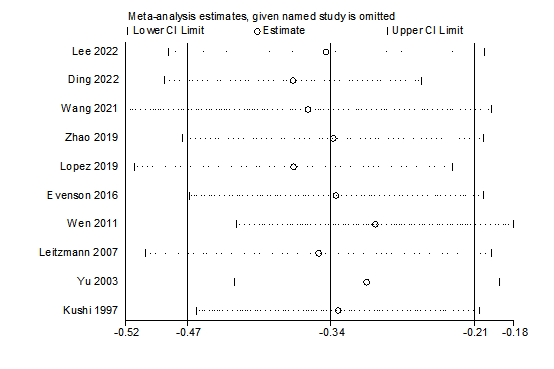
**

**eFigure 9. Sensitivity analysis for the overall summary hazard ratios for cancer mortality, omitting one study at a time.**

**
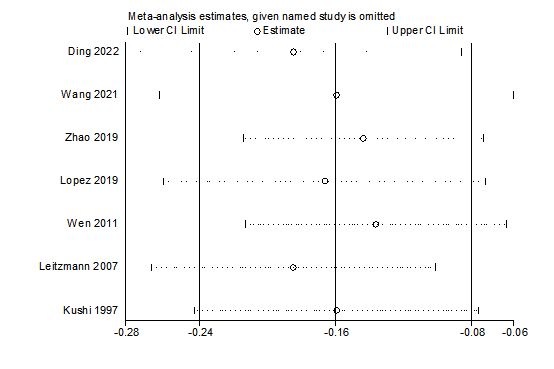
.**

**eFigure 10. Subgroup analysis of overall summary hazard ratios for all-cause modality**

**
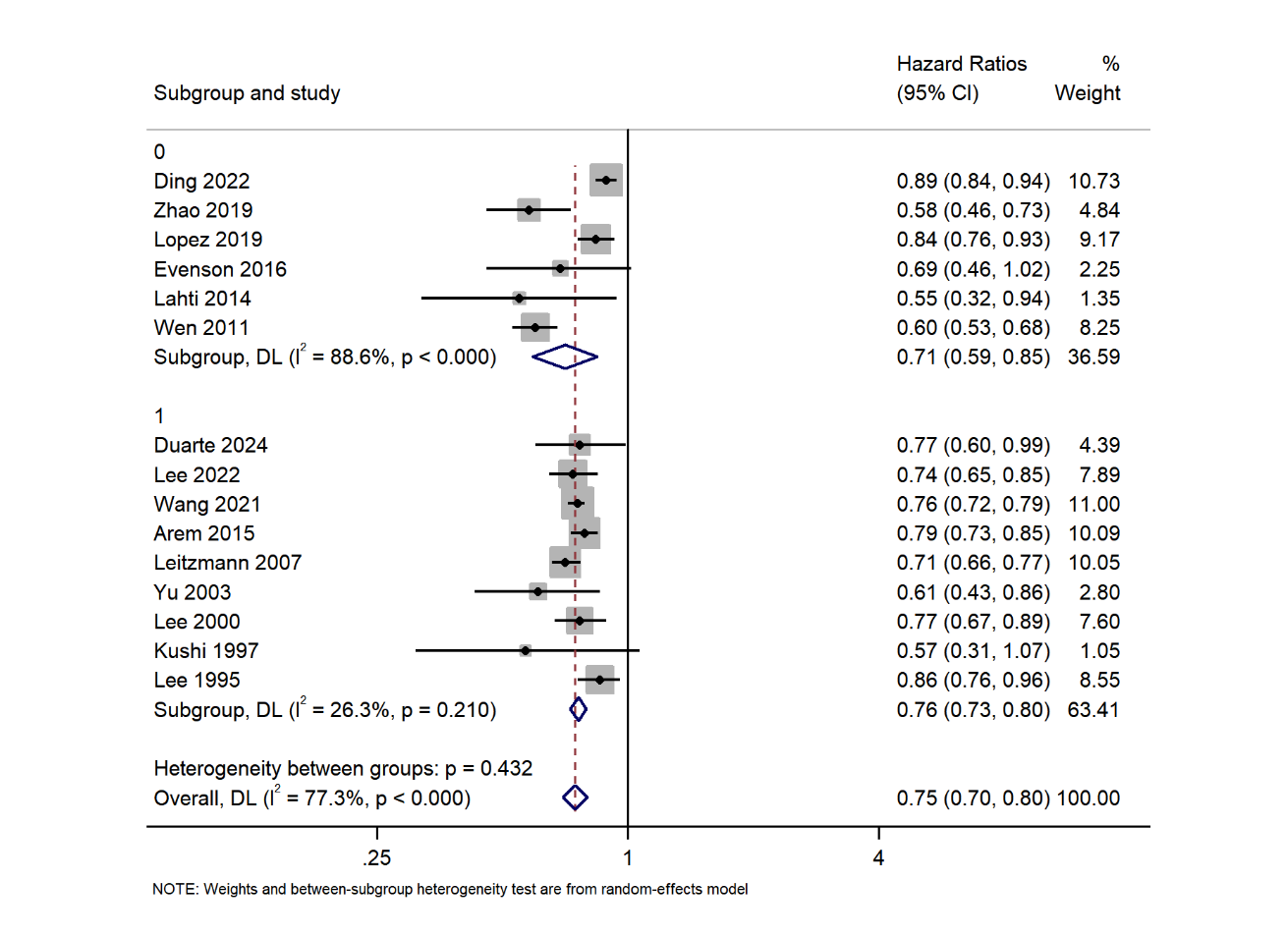
**

**eFigure 11. Subgroup analysis of overall summary hazard ratios for CVD modality**

**
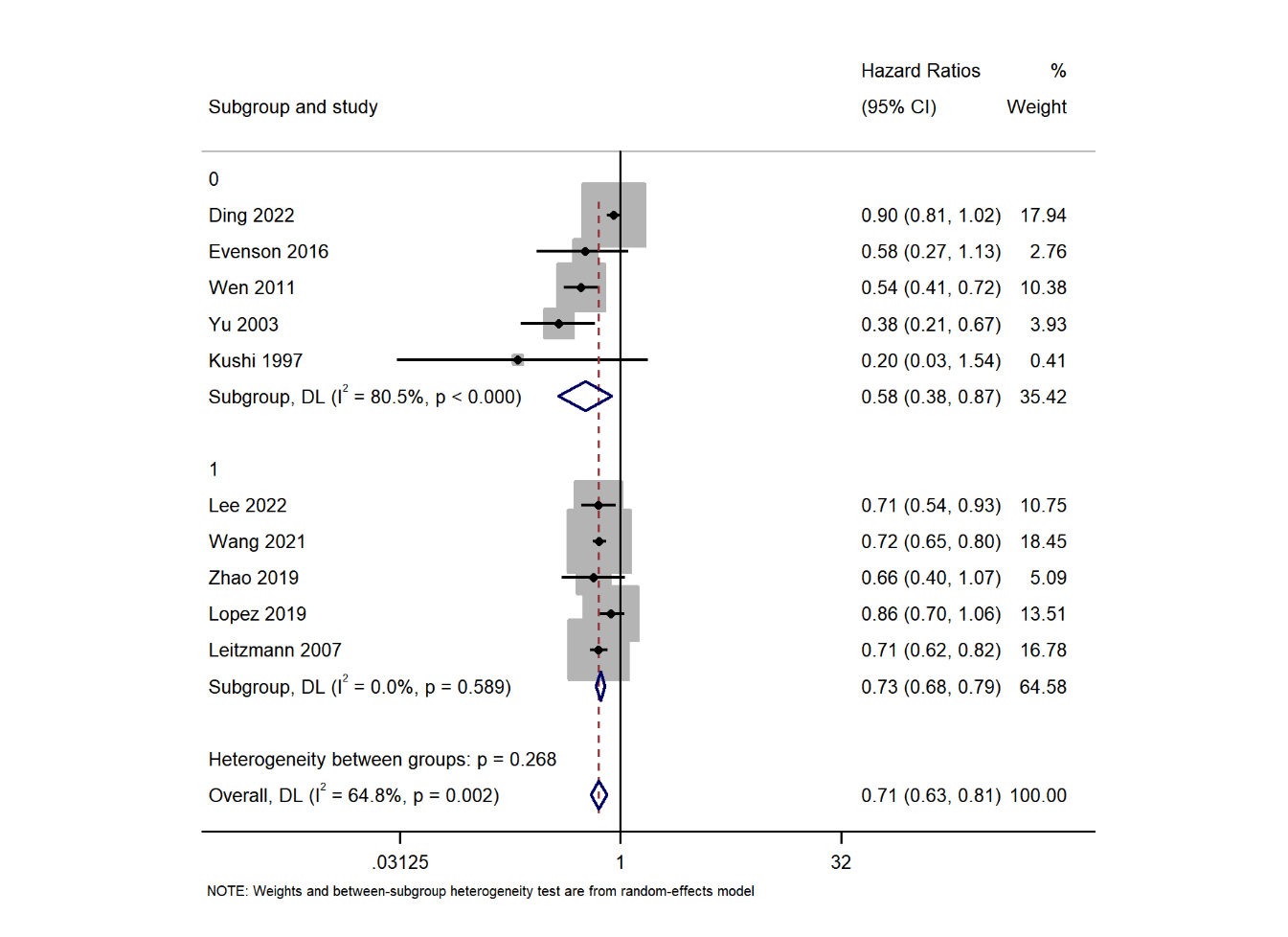
**

**eFigure 12. Subgroup analysis of overall summary hazard ratios for cancer modality**

**
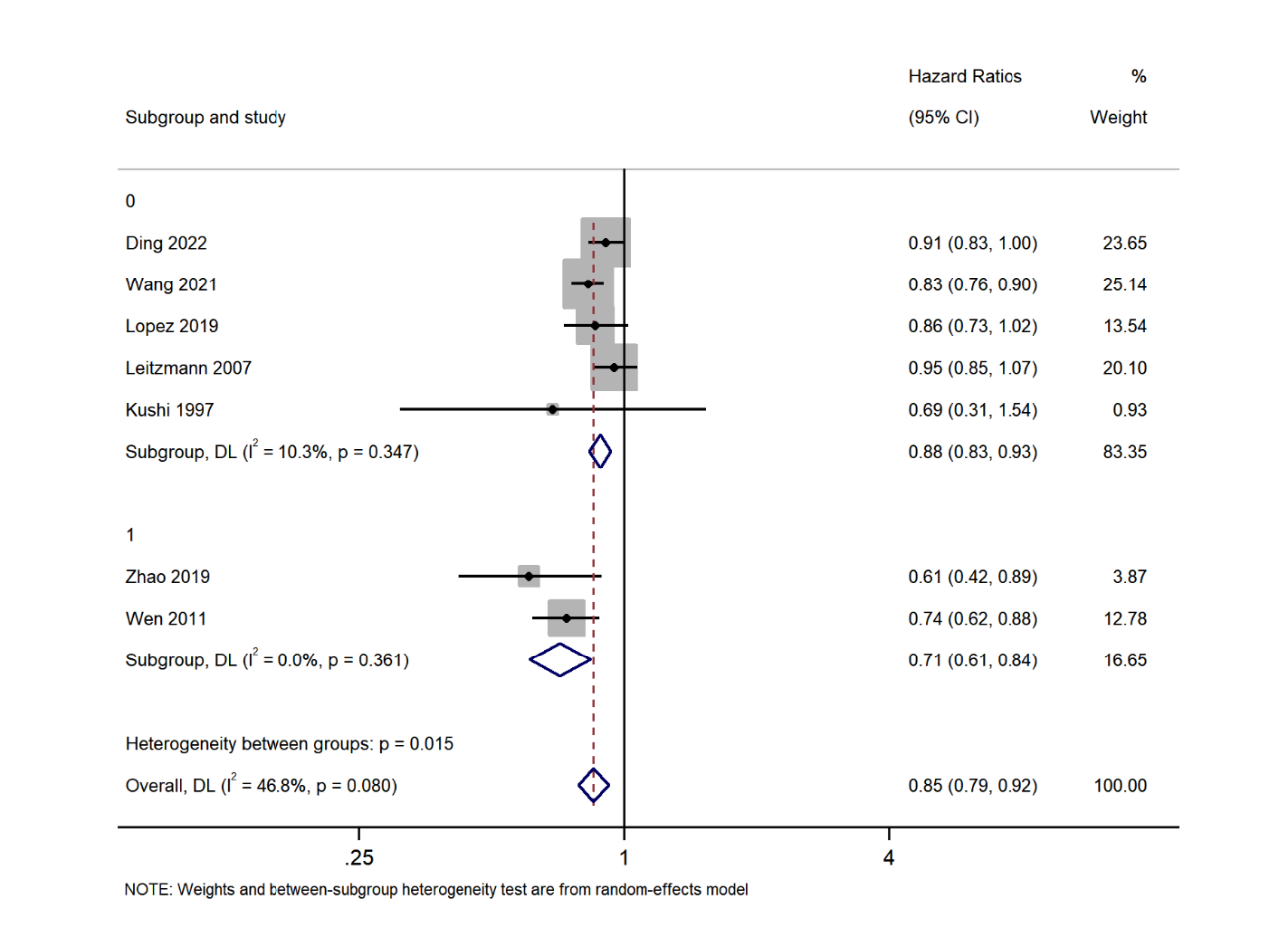
**

**eFigure 13. Funnel plot for the linear association of the HR for all-cause and CVD mortality risk against the standard error of the log (HR) per 10 percent increase in the proportion of VPA to MVPA**

**
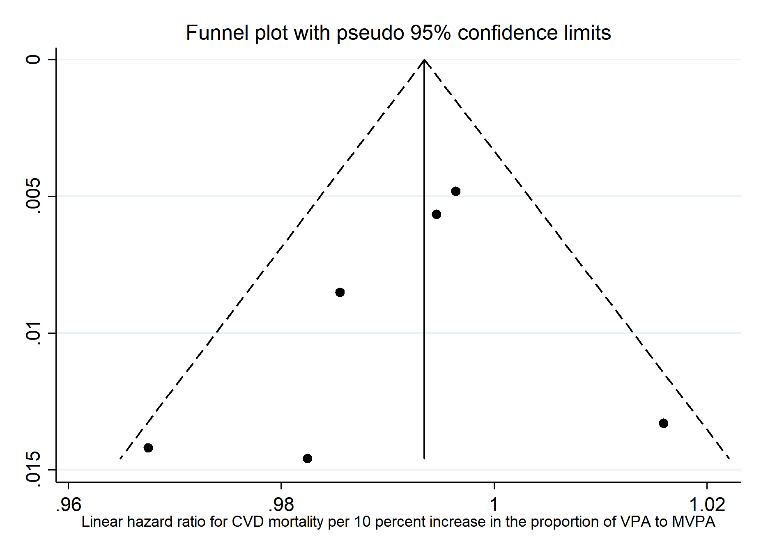

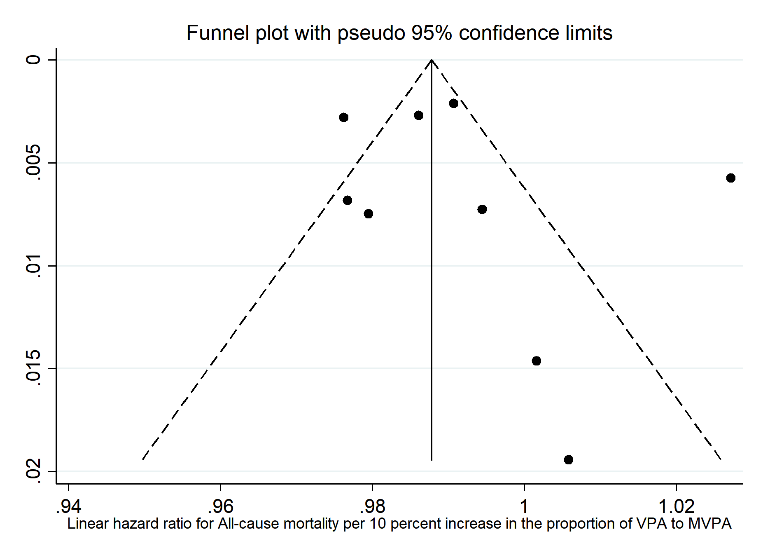
**

**
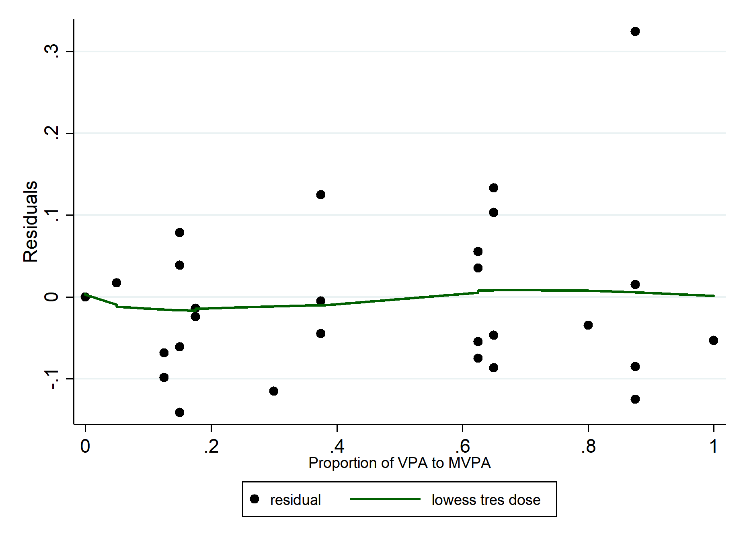

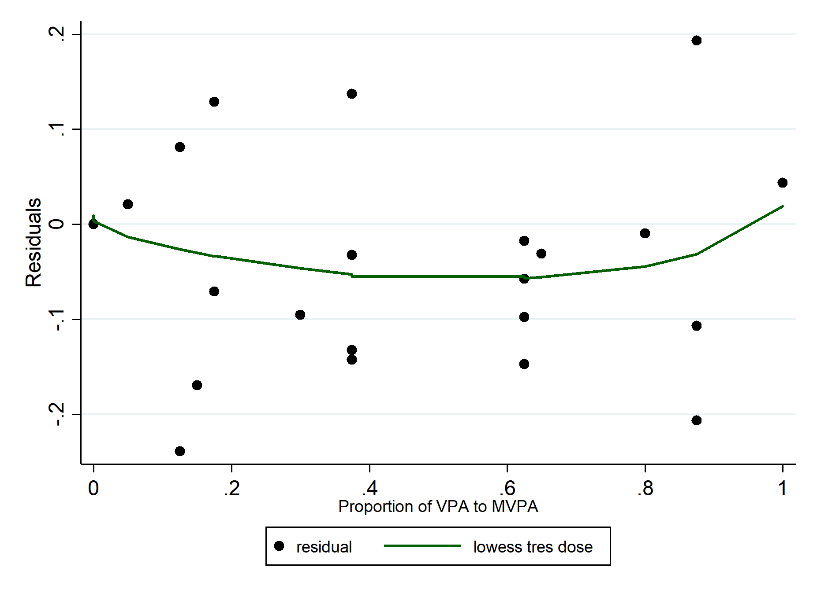
eFigure 14. Decorrelated residuals-versus-exposure plots for the proportion of VPA to MVPA dose-response analysis**

all-cause mortality, decorrelated residuals-versus-exposure plot CVD mortality, decorrelated residuals-versus-exposure plot

**
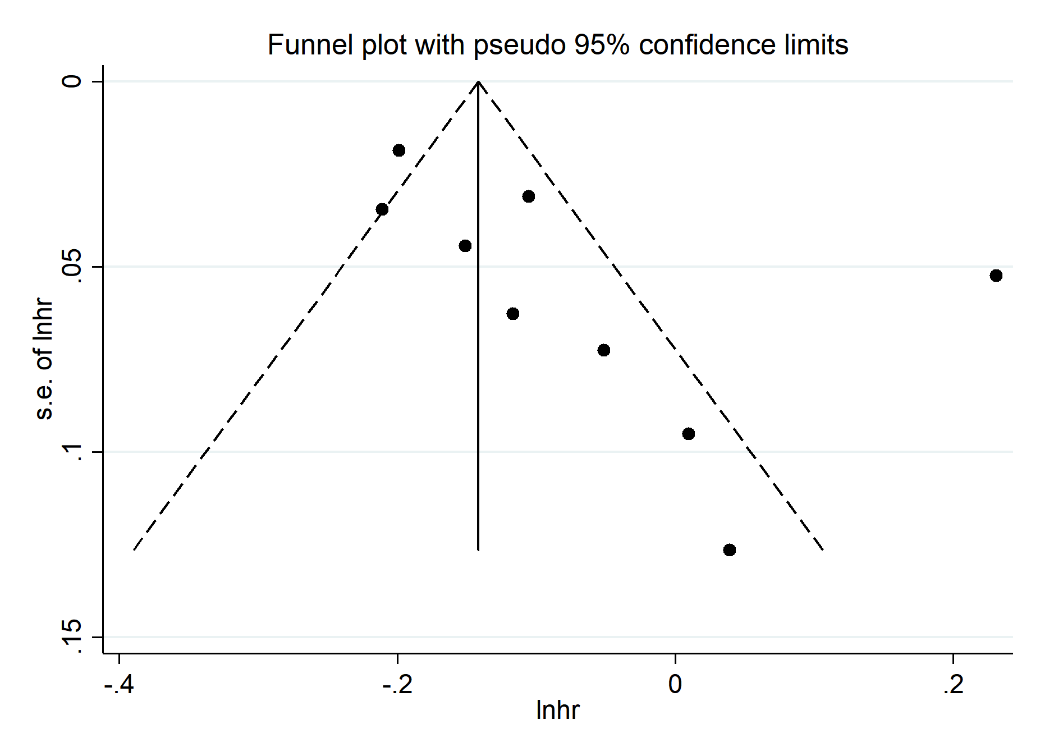
eFigure 15. Funnel plots for overall summary HRs for the highest proportion of VPA to MVPA vs. the lowest for all-cause and CVD mortality**


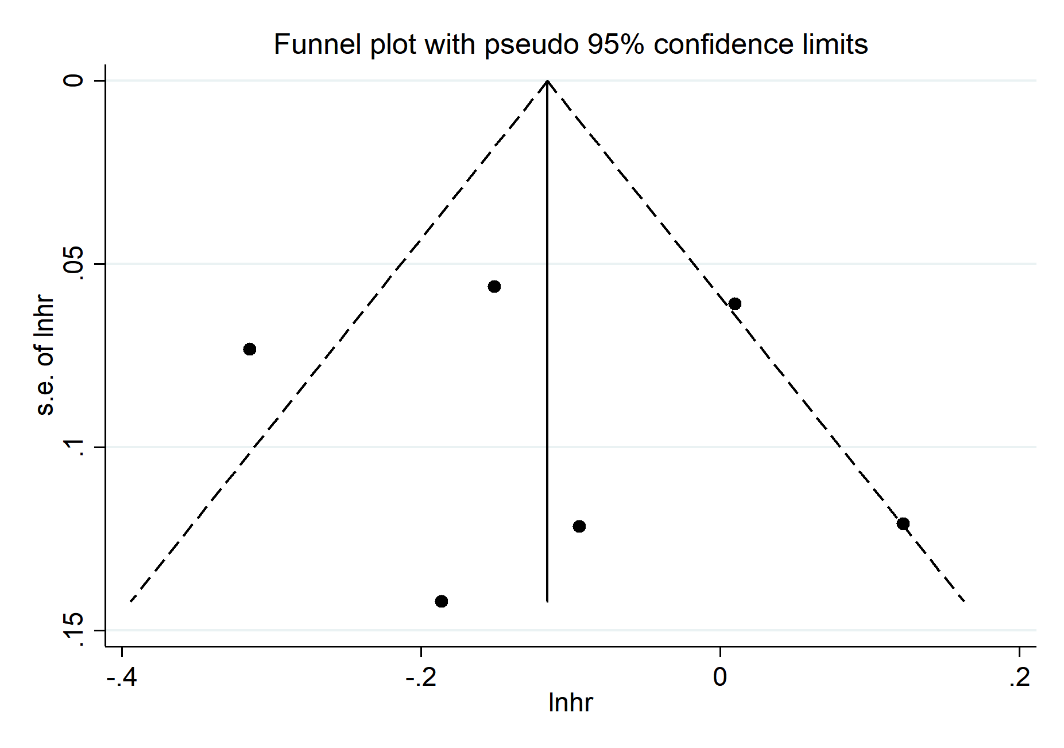
**all-cause mortality**

**CVD mortality**

**References**

1. Aune D, Greenwood D, Chan D, Vieira R, Vieira A, Rosenblatt DN, et al. Body mass index, abdominal fatness and pancreatic cancer risk: a systematic review and non-linear dose–response meta-analysis of prospective studies. Annals of oncology. 2012 Apr;23(4):843-52.

2. Duarte MA, Jr., Martinez-Gómez D, Pintos-Carrillo S, Sotos-Prieto M, Ortolá R, Rodríguez-Artalejo F, et al. Associations of physical activity type, volume, intensity, and changes over time with all-cause mortality in older adults: The Seniors-ENRICA cohorts. Scandinavian Journal of Medicine & Science in Sports. 2024 Jan;34(1):e14536.

3. Mu X, Liu S, Fu M, Luo M, Ding D, Chen L, et al. Associations of physical activity intensity with incident cardiovascular diseases and mortality among 366,566 UK adults. International Journal of Behavioral Nutrition and Physical Activity. 2022 Dec 13;19(1):151.

4. Lee DH, Rezende LF, Joh H-K, Keum N, Ferrari G, Rey-Lopez JP, et al. Long-term leisure-time physical activity intensity and all-cause and cause-specific mortality: a prospective cohort of US adults. Circulation. 2022 Aug 16;146(7):523-34.

5. Wang Y, Nie J, Ferrari G, Rey-Lopez JP, Rezende LF. Association of physical activity intensity with mortality: a national cohort study of 403 681 US adults. JAMA internal medicine. 2021 Feb 1;181(2):203-11.

6. Zhao M, Veeranki SP, Li S, Steffen LM, Xi B. Beneficial associations of low and large doses of leisure time physical activity with all-cause, cardiovascular disease and cancer mortality: a national cohort study of 88,140 US adults. British journal of sports medicine. 2019 Nov;53(22):1405-11.

7. Lopez JPR, Gebel K, Chia D, Stamatakis E. Associations of vigorous physical activity with all-cause, cardiovascular and cancer mortality among 64 913 adults. BMJ Open Sport & Exercise Medicine. 2019;5(1):e000596.

8. Ding D, Van Buskirk J, Nguyen B, Stamatakis E, Elbarbary M, Veronese N, et al. Physical activity, diet quality and all-cause cardiovascular disease and cancer mortality: a prospective study of 346 627 UK Biobank participants. British journal of sports medicine. 2022;52(20):1148-56.

9. Kikuchi H, Inoue S, Lee I-M, Odagiri Y, Sawada N, Inoue M, et al. Impact of moderate-intensity and vigorous-intensity physical activity on mortality. Medicine and science in sports and exercise. 2018 Apr;50(4):715-21.

10. Evenson KR, Wen F, Herring AH. Associations of accelerometry-assessed and self-reported physical activity and sedentary behavior with all-cause and cardiovascular mortality among US adults. American journal of epidemiology. 2016;184(9):621-32.

11. Gebel K, Ding D, Chey T, Stamatakis E, Brown WJ, Bauman AE. Effect of Moderate to Vigorous Physical Activity on All-Cause Mortality in Middle-aged and Older Australians. JAMA Internal Medicine. 2015;175(6):970-7.

12. Arem H, Moore SC, Patel A, Hartge P, De Gonzalez AB, Visvanathan K, et al. Leisure time physical activity and mortality: a detailed pooled analysis of the dose-response relationship. JAMA internal medicine. 2015;175(6):959-67.

13. Shiroma EJ, Sesso HD, Moorthy M, Buring JE, Lee IM. Do moderate‐intensity and vigorous‐intensity physical activities reduce mortality rates to the same extent? Journal of the American Heart Association. 2014;3(5):e000802.

14. Lahti J, Holstila A, Lahelma E, Rahkonen O. Leisure-time physical activity and all-cause mortality. PLoS One. 2014;9(7):e101548.

15. Wen CP, Wai JPM, Tsai MK, Yang YC, Cheng TYD, Lee M-C, et al. Minimum amount of physical activity for reduced mortality and extended life expectancy: a prospective cohort study. The lancet. 2011 Oct 1;378(9798):1244-53.

16. Leitzmann MF, Park Y, Blair A, Ballard-Barbash R, Mouw T, Hollenbeck AR, et al. Physical activity recommendations and decreased risk of mortality. Archives of Internal Medicine. 2007;167(22):2453-60.

17. Yu S, Yarnell J, Sweetnam P, Murray L. What level of physical activity protects against premature cardiovascular death? The Caerphilly study. Heart. 2003;89(5):502-6.

18. Rockhill B, Willett WC, Manson JE, Leitzmann MF, Stampfer MJ, Hunter DJ, et al. Physical activity and mortality: a prospective study among women. American Journal of Public Health. 2001;91(4):578.

19. Lee IM, Paffenbarger RS. Associations of light, moderate, and vigorous intensity physical activity with longevity - The Harvard Alumni Health Study. American Journal of Epidemiology. 2000 Feb 1;151(3):293-9.

20. Kushi LH, Fee RM, Folsom AR, Mink PJ, Anderson KE, Sellers TA. Physical activity and mortality in postmenopausal women. Jama. 1997;277(16):1287-92.

21. Lee I-M, Hsieh C-c, Paffenbarger RS. Exercise intensity and longevity in men: the Harvard Alumni Health Study. Jama. 1995;273(15):1179-84.
